# Topographical Vacuum Sealing of 3D-Printed Multiplanar Microfluidic Structures

**DOI:** 10.3390/bios11100395

**Published:** 2021-10-15

**Authors:** Benjamin Heidt, Renato Rogosic, Nils Leoné, Eduardo J. S. Brás, Thomas J. Cleij, Jules A. W. Harings, Hanne Diliën, Kasper Eersels, Bart van Grinsven

**Affiliations:** 1 Sensor Engineering Department, Faculty of Science and Engineering, Maastricht University, P.O. Box 616, 6200 MD Maastricht, The Netherlands; renato.rogosic@maastrichtuniversity.nl (R.R.); thomas.cleij@maastrichtuniversity.nl (T.J.C.); hanne.dilien@maastrichtuniversity.nl (H.D.); kasper.eersels@maastrichtuniversity.nl (K.E.); bart.vangrinsven@maastrichtuniversity.nl (B.v.G.); 2 Aachen-Maastricht Institute for Biobased Materials, Faculty of Science and Engineering, Maastricht University, P.O. Box 616, 6200 MD Maastricht, The Netherlands; nils.leone@maastrichtuniversity.nl (N.L.); jules.harings@maastrichtuniversity.nl (J.A.W.H.); 3 Department of Biomedical Engineering, Faculty of Medicine, Eberhard Karls University Tübingen, 72770 Tübingen, Germany; eduardo.bras@nmi.de

**Keywords:** microfluidics, 3D printing, vacuum forming, topographical vacuum sealing, TOVAS

## Abstract

We demonstrate a novel way of creating three-dimensional microfluidic channels capable of following complex topographies. To this end, substrates with open channels and different geometries were 3D-printed, and the open channels were consecutively closed with a thermoplastic using a low-resolution vacuum-forming approach. This process allows the sealing of channels that are located on the surface of complex multiplanar topographies, as the thermoplastic aligns with the surface-shape (the macrostructure) of the substrate, while the microchannels remain mostly free of thermoplastic as their small channel size resists thermoplastic inflow. This new process was analyzed for its capability to consistently close different substrate geometries, which showed reliable sealing of angles >90°. Furthermore, the thermoplastic intrusion into channels of different widths was quantified, showing a linear effect of channel width and percentage of thermoplastic intrusion; ranging from 43.76% for large channels with 2 mm width to only 5.33% for channels with 500 µm channel width. The challenging sealing of substrate ‘valleys’, which are created when two large protrusions are adjacent to each other, was investigated and the correlation between protrusion distance and height is shown. Lastly, we present three application examples: a serpentine mixer with channels spun around a cuboid, increasing the usable surface area; a cuvette-inspired flow cell for a 2-MXP biosensor based on molecular imprinted polymers, fitting inside a standard UV/Vis-Spectrophotometer; and an adapter system that can be manufactured by one-sided injection molding and is self-sealed before usage. These examples demonstrate how this novel technology can be used to easily adapt microfluidic circuits for application in biosensor platforms.

## 1. Introduction

Microfluidics is a field of growing importance for biosensing, given the enormous capabilities it offers in terms of sample preparation, sample manipulation and reagent storage in minimal form factors that are especially advantageous in point-of-care scenarios. The field of microfluidics started out on the shoulders of micro-electromechanical systems (MEMS) manufacturing, where it applied microchip fabrication technology to create microfluidic channels [1]. MEMS techniques like photolithography remain—despite requiring a clean room and a work-intensive manufacturing process—the gold-standard up until today. Many advances have been made to lift the restrictions of clean-room fabrication [2,3,4], such as replacement of the vapor-deposited metal-masks with masks that are printed with a conventional laser printer on a transparent surface [5,6]. This method enables the user to skip the mask manufacturing with its many in-between steps. Furthermore, some developments forego the use of a mask altogether and use printing technologies to directly print the master mold [7,8,9]. Other approaches go even further and replace the complete lithographic process with new means of production, for example, the use of shrinkable polymers as a substrate [10,11]. Another approach of cleanroom-free manufacturing is lab-on-a-foil (LoaF) [12]. Here, channels are either cut [13,14] or hot-embossed directly into polymer films [15,16,17]. This approach also offers the benefit of being compatible with flexible substrates. While most other microfluidic channels are created on a rigid surface, lab-on-a-foil systems can bend, though this is still limited by the rigidness of the foil used. Vacuum-forming technology is already utilized in LoaF contexts, but contrary to our novel approach, it is only used in the creation of channels via molding, which are then de-molded and sealed in a later step [18,19,20,21]. 

More recently, 3D printing has become increasingly popular for the cleanroom-free fabrication of microfluidic structures, driven by the decrease in price and increase in resolution of available 3D printers within the last 10 years [22]. While their resolution, for the most part, still cannot match classical clean-room photolithographic techniques, they excel in terms of ease of use and rapid prototyping. Another benefit of 3D printing is the capability to create three-dimensional channels, allowing for new applications and form factors compared to the usually monoplanar traditional methods. 

The two most common methods for 3D printing are fused deposit modelling (FDM), where a thermoplastic filament is molten in a heated nozzle and added layer-by-layer to create a three-dimensional construct, and SLA-printing, where a liquid photopolymer is cured by exposition to UV light. The light is either directed by a screen, displaying the whole layer (based on DLP or LCD-screen technology, both summarized as mSLA printing), or by a laser that ‘writes’ the structure into the polymer (laser SLA printing) [23]. 

While commercial FDM printers specialized in microfluidics are available and techniques like the ESCARGOT method open up interesting capabilities [24], FDM printers still suffer from a lower resolution compared to SLA printers. 

Therefore, SLA printing is often the method of choice to produce small features. For the direct manufacturing of microfluidic channels, it is either possible to create closed channels embedded inside the printed device or as groves on the surface. While the creation of closed channels gives more possibilities in terms of three-dimensional creation, it is difficult to produce them in small sizes with common desktop printers, as the smaller the channel size, the more likely it is that resin gets trapped inside and is cured by stray light during printing, resulting in clogging of the channel [25]. Therefore, the creation of surface channels is vastly more reliable and smaller channel sizes can be achieved, but it opens up another problem: the channel needs to be sealed to be of use. 

On monoplanar substrates, this is achieved by the use of a sealing plane that is attached to the plane with the open channels by either plasmabonding, thermobonding, ultrasonic welding or simply the use of adhesive tape. This plane creates the outer limit to the open channels and seals them. However, this negates the benefit of 3D printing to create microfluidic devices in new form factors, as sealing channels this way is challenging and time consuming for difficult topographies that are multi-planar. 

In this work, we show an easy and cost-effective way to seal open channels on challenging topographic surfaces using a vacuum-forming approach called topographical vacuum sealing (TOVAS). This technique can be used in combination with 3D-printing (or other nonplanar methods like micro-milling), to create complex topographic devices in form factors that were previously impossible. We benchmark the capabilities of the new method in terms of the topography that can be achieved and present three different use cases of the new method.

## 2. Materials and Methods

**Figure 1:**
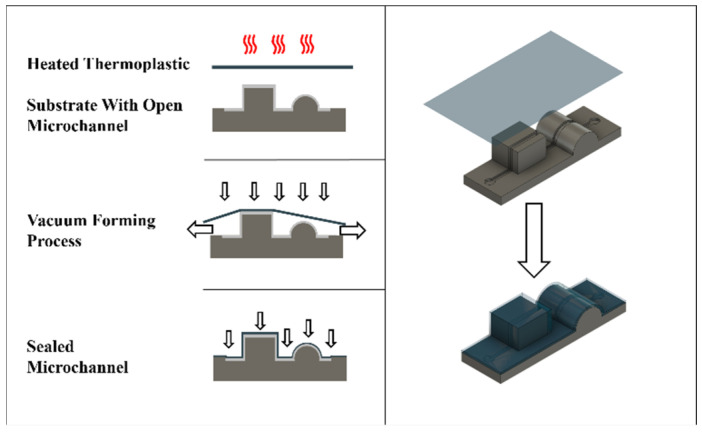
The fabrication process of topographical vacuum sealing. A thermoplastic is heated above its glass transition temperature and stretched over the topographic substrate. The vacum alignes the thermoplastic to the surface of the substrate, while the microchannels remain free due to their small size.

**Figure 2:**
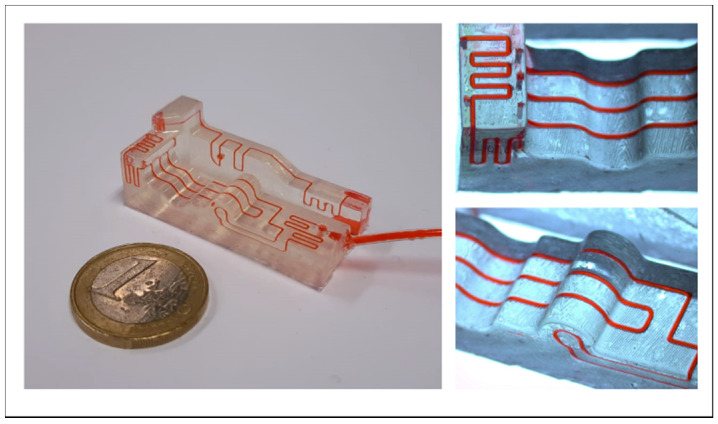
Testing device covering several challenging geometries and displaying the flexibility of the method.

The new approach consists of a multi-planar substrate, which can be 3D printed, injection molded, hot embossed or micro-milled. The substrate displays open microfluidic channels on its surface. The channels are sealed by using a low-resolution vacuum-forming process that stretches a heated thermoplastic over the multi-planar substrate. The thickness of the thermoplastic ensures that the process remains ‘low resolution’ and only follows the macro-topography instead of also intruding into the microchannel, as is done by vacuum forming of the channels directly. Figure 1 shows the manufacturing process and Figure 2 shows a testing structure with various challenging geometries that can be covered.

### 2.1. Printing and Vacuum Forming

Several testing structures were designed using Fusion 360 (Version 2.0.9719, Autodesk, San Rafael, CA, USA) and constructed via 3D printing, using a Formlabs Form2 SLA Printer (Formlabs, Somerville, MA, USA). All devices were printed with clear resin V4 (FLGPCL04, Formlabs, Somerville, MA, USA). After printing, the devices were washed for 20 min in isopropanol (BOOM, Technical Grade, Fischer Scientific, Landsmeer, The Netherlands) in a Form Wash (Formlabs, Somerville, MA, USA) and postcured for at least 20 min at 60 °C in a Form Cure postcuring machine (Formlabs, Somerville, MA, USA). In devices where geometry makes the resin difficult to wash out, compressed air was used to clean them post-washing, and small holes were flushed with isopropanol to remove the remaining resin. 

After postcuring, a layer of spray adhesive (UHU Spray glue-UH46740) was evenly applied over the whole bonding surface of the substrate. A sheet of 500 µm-thick Polyethylene Terephthalate Glycol (PETG) (Mayku Cast Sheets, Mayku, London, UK) was placed into a vacuum former (JT-018, DIFU Vacuum Former). The machine used is a low-cost device with fixed heating and vacuum settings; it features a movable tray that holds the thermoplastic and is able to be fixed in two positions: In the heating position, the tray is moved upward directly under the heating element, where it is heated. To start the vacuum forming process, the tray is lowered onto the substrate, which is located on a grid above the vacuum pump. The measured variable to ensure repeatability with this type of system is the plastic deformation of the thermoplastic. As the thermoplastic is continuously heated, it surpasses its glass transition temperature and starts to deform; the deformation of the thermoplastic is visible as a clear bulge starting to droop beneath the holding tray. The vacuum-sealing process was started when the thermoplastic reached 1 cm under the holding tray, which takes 1.5 min of heating and corresponds to a temperature of 180 °C, as measured with a thermal camera (Akzon HT-18, e = 0.95). The thermoplastic was pulled over the substrate by lowering the tray into the vacuum-forming position and the vacuum pump of the device was activated to introduce a negative pressure measured at −165 mbar for 4 s. This aligned the hot thermoplastic to the substrate surface and thus sealed the prepared topography. An image of the machine and its parts can be found in Supplementary Figure S3. To promote adhesion, the device was left to cure at room temperature and only used after 24 h. Every test device was printed and tested in triplets.

### 2.2. Testing Devices

#### Angle Leakage

Three types of 3D prints were manufactured to test the channel integrity when traversing different angles. The tested angles were 90°, 120°, 150°, 210°, 240°, 270° and 330°. To better visualize leakage, two control channels were fabricated next to the fluid-carrying channel. The fluid channel was flushed with water containing red food coloring. The flushing speed was varied from 1 to 10 mL/min in 1 mL/min increments. If a channel shows leakage, the control channels will fill and show clear red indicators. The channel size and depth of fluid-carrying and control channels was 500 μm and the distance between them 466 µm. To test if the sealing for 90° angles could be improved, an additional type of test-structure was prepared with three 90° angles and different degrees of filleted edges, with 1 mm, 1.5 mm and 2 mm fillet-radii, respectively.

### 2.3. Maximum Working Pressure

To test the maximum pressure which the substrate-thermoplastic interface can withstand, three test structures were prepared. The structures featured a 500 µm dead-end channel flanked by two open-ended 500 µm control channels at both sides. The test structures were prepared with different topographies: one monoplanar (flat) structure; one prism-shaped topography, with angles of 135° (base of prism) and 270° (top of prism); a cube structure with a 90° angle, containing a 1 mm fillet (base of cube) and a 270° angle (top of cube). The structures featured a small reservoir for liquid and a LUER slide adapter to connect the device to a pressure line via a hose. Air pressure was used to force a column of water containing red food coloring out of the small reservoir into the dead-ended channel, increasing the pressure in the process. To prevent leakage from the adapter, it was glued in place for every device using PSMT3 Superglue (Pattex, Duesseldorf, Germany).

### 2.4. Column Separation

To assess how sensitive the methodology is to the spacing between two large protrusions, test structures were created with varying heights and distances between two adjacent protruding columns. If two columns were too close to each other and/or too high, the thermoplastic would have difficulties covering the space in-between the protrusions. This would limit the form factors of the methodology. Therefore, we created test structures with columns of various heights (1–6 mm), spaced at different distances from each other (2–6 mm). The devices were prepared and sealed with the aforementioned method and measured using a digital caliper (DIGI-MET 1320 417, Helios Preisser, Gammertingen, Germany).

### 2.5. Channel Intrusion

When the thermoplastic is aligned to the substrate macro-structure, it will slightly intrude into the microchannel. To quantify to what extent this occurs, a structure with different channel widths was printed. The thermoplastic was aligned as described but omitting the spray glue to be able to release the formed thermoplastic. The thermoplastic was then used as a mold and filled with Polydimethylsiloxane (PDMS, Sylgard 184) with a ratio of 1:10 curing agent to monomer. After degassing, it was cured for 1.5 h at 70 °C to create an imprint of the intrusions. This PDMS imprint was removed from the thermoplastic and cut cross-section. A Leica S6E microscope with a Leica DFC290 HD digital camera was used to take pictures, the prints were aligned to account for the viewing angle and the pictures were quantified using imageJ. Translation to PDMS was performed as the thin thermoplastic might have deformed while being cut; this was prevented by using the thicker PDMS imprint that is more resistant to deformation and is easier to observe via a microscope.

## 3. Results

### 3.1. Angle Leakage

**Figure 3:**
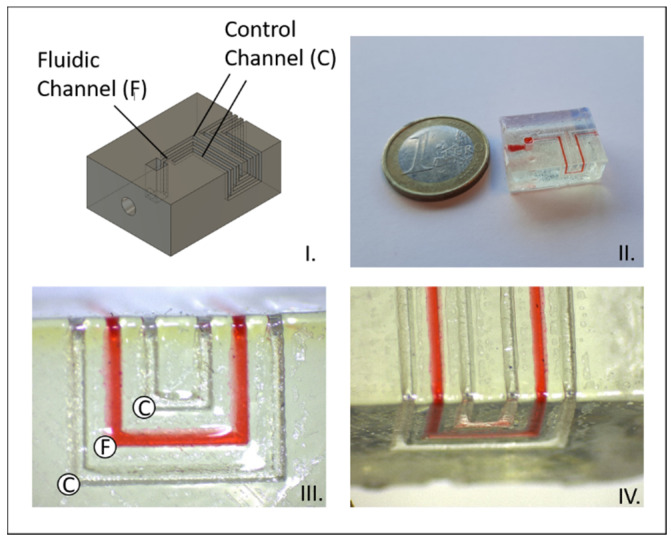
(**I.**) Rendering of a basic example of a testing chip with two crossings of a 270° angle. The control channels are flanking the fluid channel and turn red if they fill due to leakage. (**II.**) Resulting 3D-print carrying water colored with red food coloring. (**III.**,**IV.**) Close-up of the angle crossing with control channels (C) and the fluid carrying channel (F).

Figure 3 shows the basic function behind the test structures. The basic test structure consists of a 500 µm fluid-carrying channel that is aligned in-between two identical 500 µm empty control channels. The adjacent pair of channels serve as visual controls to quickly identify the possibility of a leakage during the liquid injection, as they would be rapidly filled with the colorant. In Figure 3, a 270° crossing without leakage is shown.

**Figure 4:**
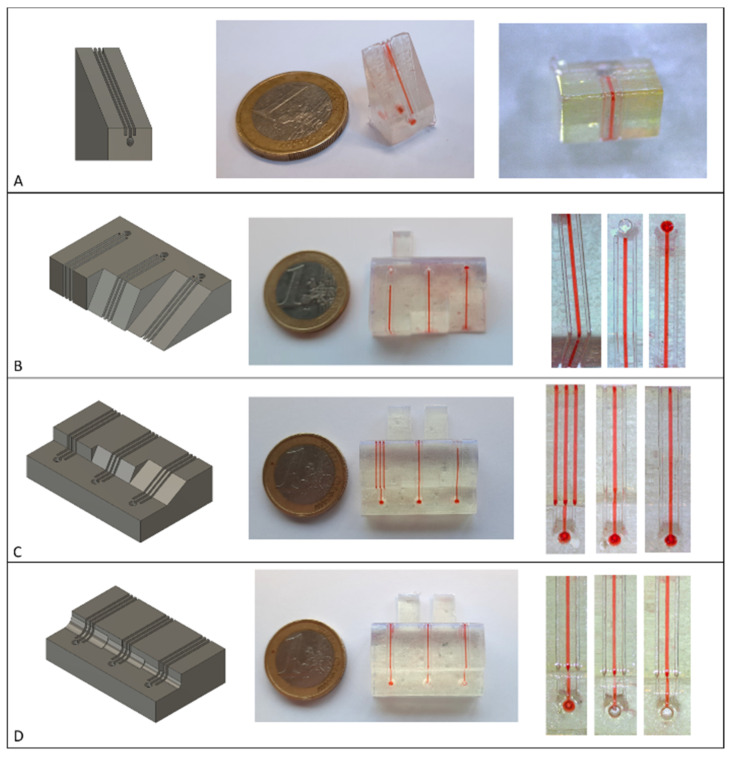
Different structures for testing channel integrity while traversing different angles. Left: Rendering of the structure. Middle: Completed print sealed with the topographical vacuum forming method. Right: Magnified channels. (**A**) 330° angle. (**B**) From left to right: 270° 240° and 210° angles. (**C**) From left to right: 90°, 120° and 150° angles. (**D**) From left to right: 90° angles with 2 mm, 1.5 mm and 1 mm radius fillets.

The different test structures created to test the methodology are shown in Figure 4 The control channels remained free for all tested flow rates for the channels traversing the angles 330°, 270°, 240°, 210°, 150° and 120°. Only the 90° angle leaked, as shown by the filled control channels. All 90° crossings with filleted edges remained leakage-free, proving that applying a fillet over an angle improves the thermoplastic contact and sealing.

### 3.2. Maximum Working Pressure

**Figure 5:**
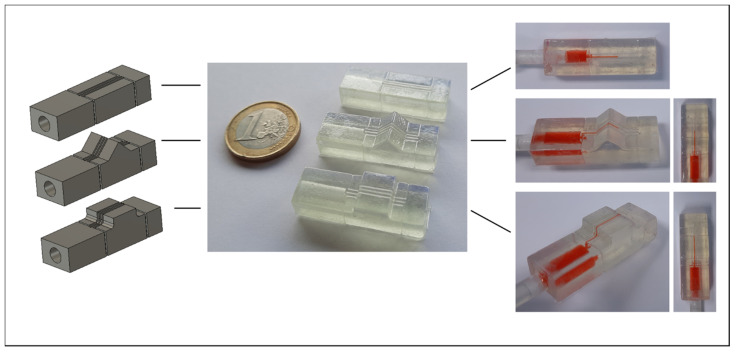
Different structures for the maximum pressure test. Left: Rendering of the structures. Middle: Completed print, sealed with the topographical vacuum forming method. Right: Pressurized channel with water. Top line: Monoplanar structure. Middle line: Prism-shaped structure with 135° and 270° angles. Bottom line: Cube extrusion with 90° angle, 1 mm fillet and 270° angle.

In the maximum pressure test, a 6-bar pressure was achieved without any leakage in all the created test structures. This was the maximum pressure available in the laboratory. Figure 5 shows the different structures, with the water column forced into the middle channel by the applied pressure.

### 3.3. Column Separation

**Figure 6:**
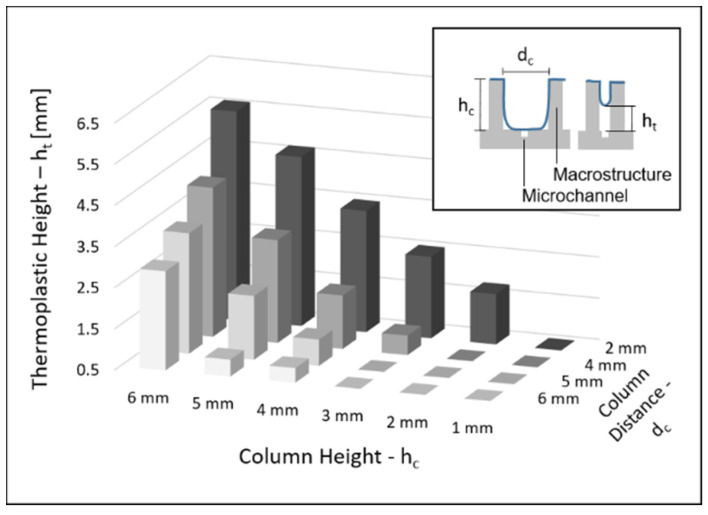
Relationship between column height, column distance to each other and the height of the thermoplastic spanned in-between the columns. Heights at 0.5 mm made contact with the substrate, as this is the thickness of the thermoplastic sheets. The complete dataset as a 2D graph is available in the supplementary information.

Figure 6 shows the height of the thermoplastic stretched between the columns relative to the bottom of the structure (in other words, how far down the thermoplastic was able to stretch between the two protrusions). The height of the thermoplastic was 0.5 mm, which was chosen as a cut-off value since below this distance, the ground in-between the columns can be considered sealed, which was optically confirmed for every case. The highest hanging thermoplastic is seen in the highest columns that are closest together. For 6 mm- to 4 mm-high columns, no thermoplastic was able to cover the space in-between. For 3 mm-tall columns, the space in-between was sealed when the columns were 6 and 5 mm apart. For 2 mm-tall columns, all distances connected to the ground except in-between the 2 mm-distant columns. Lastly, the 1 mm height did connect completely even for the columns only 2 mm apart. In general, the further apart and lower the columns were, the easier it was for the thermoplastic to align with the ground in-between.

### 3.4. Channel Intrusion

**Figure 7:**
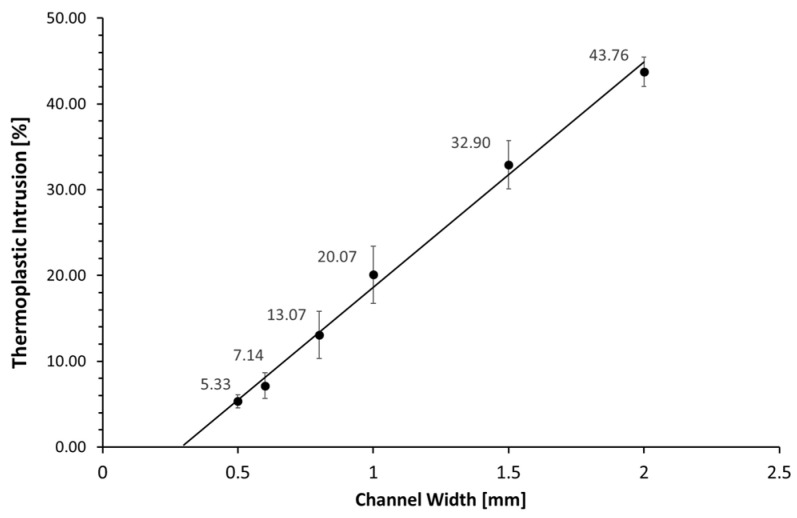
Thermoplastic intrusion into channel as percentage of channel width.

Figure 7 shows the intrusion of thermoplastic into the channels as a percentage of channel width. The 2 mm-wide channels showed the largest intrusion into the channel; with 880 µm, the intrusion is almost half the channel size (43.76%). Channel intrusion decreased with smaller channel sizes. The 1 mm-wide channel only had an intrusion of 200 µm (20.07%) and the 0.5 mm wide channel only had an intrusion of 30 µm (5.33%). The general trend shows that the smaller the microchannel, the less channel intrusion seems to be a problem.

### 3.5. Application: Roundabout Serpentine Mixer

**Figure 8:**
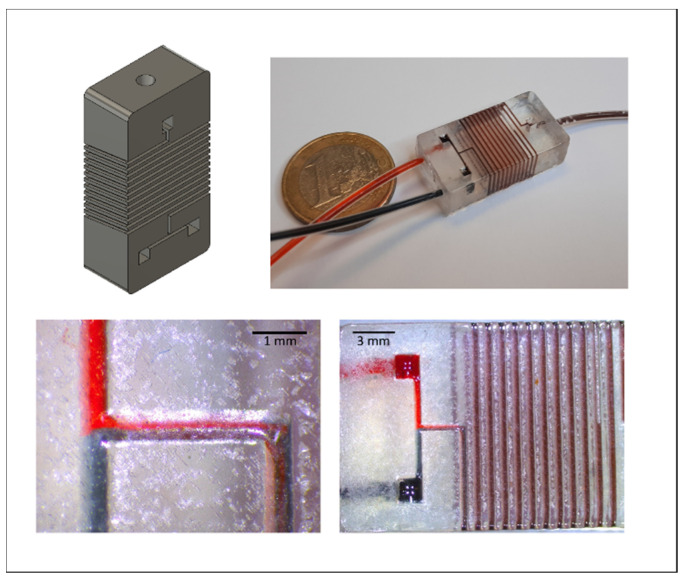
Meander structure as a serpentine mixer going the whole way around a cube, increasing the usable space.

The developed methodology allows for the fabrication of a whole new range of applications. In this experiment, we used it to create a serpentine mixer that was formed around a cuboid, with channels wound 12 times around all four sides of the mixer. To illustrate the concept, the mixer was flushed with water containing blue and red food coloring, respectively, at a flow rate of 1 mL/min (Figure 8). The methodology enables users to use substrates more efficiently and increase the length of the mixer pathway without changing the volume of the resulting device. It is also possible to extend the concept toward heating-cooling systems by micro-milling similar channels into metal substrates and covering the channels with the same technique, thus drastically increasing the contact area between the liquid and the metal substrate. Here, the glass-transition temperature has to be taken into account (75–80 °C for PETG) and serves as an upper limit of the application. While this can be too low for applications like chemical synthesis, it is still useful for other use-cases such as, for example, biosensing with the heat-transfer method, where measurements are often conducted at 37 °C [26,27,28]. Furthermore, in a similar system, the substrate could be used as electrodes for electrochemical measurements.

### 3.6. Application: UV-Vis Cuvette for Colorimetric Biosensing

**Figure 9:**
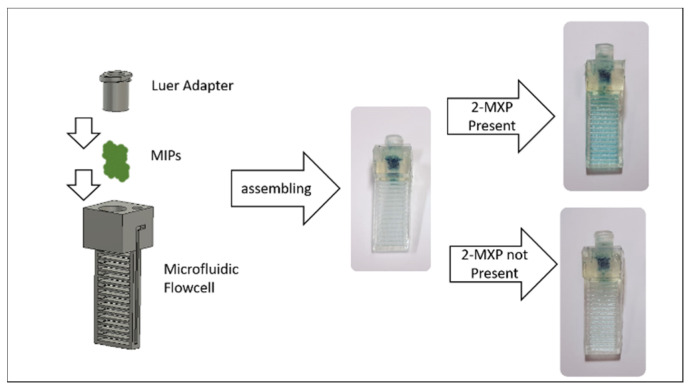
A microfluidic ‘cuvette’ with the same width and depth as a cuvette; this device can be used in any standard UV/Vis spectrophotometer to conduct online measurements. It is filled with molecular imprinted polymers loaded with a malachite green, which is released on contact with the drug 2-MXP.

**Figure 10:**
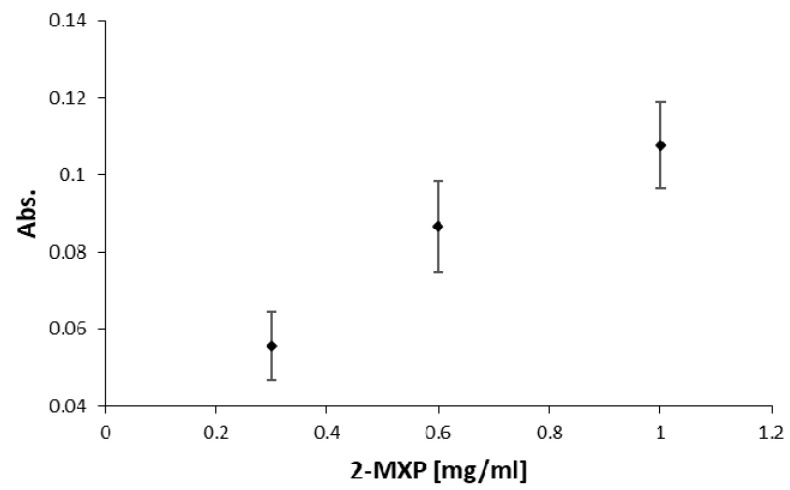
Absorbance measurement at 617 nm for the cuvette-shaped microfluidic structure, flushed consecutively with 0.3, 0.6 and 1 mg/mL 2-MXP solution.

To further illustrate the application potential of the methodology, we developed a device for optical sensing experiments. The device was created with the same width and height as a standard spectrophotometer cuvette, enabling online optical measurement of processes that are compatible with every standard UV/Vis spectrophotometer. The device incorporates molecular-imprinted polymers (MIPs) as synthetic receptors for drug detection. The MIPs were imprinted with the designer drug molecule 2-methoxiphenidine (2-MXP). More experimental details on the synthesis procedure can be found in earlier work [29]. After imprinting, the template molecules were washed out and the remaining MIP cavities were loaded with a dye (malachite green). Previous work has demonstrated that when these dye-loaded MIPs come in contact with the target analyte in solution, the loaded dye is displaced by the target molecule and set free in the surrounding solution, leading to a visibly observable color reaction that can be quantified with a spectrophotometer [29,30]. In this experiment, we integrated the MIP-based assay and the cuvette into a singular device, which we used to do a substrate displacement colorimetry (SDC) experiment with a commercial Shimadzu UV-1800 spectrophotometer. The device was loaded with 30 mg of dye-loaded MIP powder, and solutions containing 0.3, 0.6 and 1 mg/mL MXP were flushed through the Luer adapter into the cuvette (Figure 9). The absorption values obtained from these experiments, performed in triplicate for each experiment, were used to construct a basic dose-response curve, which is shown in Figure 10. These results indicate that the methodology could be used to produce low-cost, user-friendly consumables for SDC (and other biosensor applications) on a large scale. The thermoplastic used is largely optically transparent in the visible range and shows absorption only in the UV range under 350 nm, which makes it highly usable for colorimetric assays. To further account for the optical properties, a blank with di-water was used.

### 3.7. Application: Luer-Adapter

**Figure 11:**
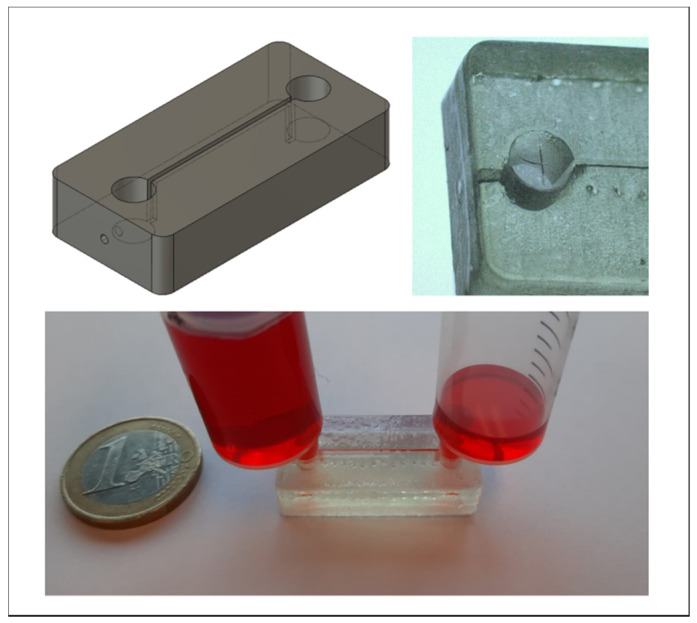
Channel with 3D-printed Luer-Adapter. The channel traverses completely on the upper surface and thus can be easily injection molded.

Figure 11 shows a 3D-printed channel with two Luer inlets at both sides. The channel traverses from the bottom of the Luer adapter to the upper side of the channel. Since the critical components are completely on the surface and facing the same direction, the design lends itself to fabrication methods other than 3D printing, for example, such a part can easily be injection-molded since the mold only has to have features at one side. After topographical vacuum sealing, the channels, as well as the Luer-adapter, are closed, isolating the system from the environment. Weakening the thermoplastic above the Luer-port, by for example, scratching the surface, enables syringes to easily puncture the system, giving easy access to the Luer port when needed, while isolating it beforehand. The thermoplastic slightly intrudes into the Luer adapter, thus creating a reliable seal at the edge from the internal Luer-adapter surface to the top surface of the substrate, something that would be difficult to achieve by sealing with adhesive tape alone.

## 4. Discussion

We demonstrated a novel method for the creation of topographical microfluidic channels and gave several demonstrations of how the newly possible form factors of the devices can be applied to create microfluidic structures for biosensing applications. The possible applications span a wide range of different fields, for instance, creating devices for healthcare, food safety and even education [31].

The method works reliably for a wide array of shapes; the angles covered the range from 90° to 330° with only the 90° angle showing leakage. Applying a fillet to the 90° angle prevented the leakage, showing that the thermoplastic was most likely unable to intrude and adhere to the tight edge. A rounding of the edge should be possible in almost all designs, eliminating the problem. The column separation experiment showed the relationship between the height and distance of protrusions and the ability of the thermoplastic to align to the full structure. Here, high protrusions in close proximity were more difficult to align to, compared to short protrusions at greater distances from each other. Given that the alignment is dependent on the plasticity of the material, an increase in temperature should improve the alignment of the thermoplastic onto the substrate. A similar improvement might be achieved with an increase in negative pressure or the use of a thermoplastic with a lower glass transition temperature such as polylactic acid. In particular, substrates with difficult geometries, like several protrusions that are high and/or in close proximity to each other, might benefit from these changes. However, increasing the plasticity may also lead to greater unwanted intrusion into the microchannel. Conversely, to minimize the microchannel intrusion, lowering the temperature and vacuum settings—as well as using a thermoplastic with a higher glass transition temperature such as polystyrene or polycarbonate—might achieve better results. This would be especially helpful for substrates that have a simple surface geometry, with low and few protrusions but larger channels, since here, the surface alignment will be less of a problem than the channel intrusion. Therefore, even though the parameters used in this study are widely functional, the process can still be further optimized and adapted to different applications and substrate geometries, to find the best balance between the channel intrusion that was shown in Figure 7 and the macro-structure alignment shown in Figure 6, on a case-by-case basis.

The pressure stability of the substrate-thermoplastic interface was measured at up to 6 bar, which is way above that necessitated by most microfluidic applications. This was also the maximum pressure available in the laboratory and thus the actual value is most likely higher. However, the maximum pressure the device can handle is also dependent on the substrate geometry, and while it was tested with different structures, this may be lower in substrates with more challenging features or substrates containing several weak points. Therefore, the measured value is still to be seen as a value under ideal circumstances.

One of the main benefits of the presented method is its ease of use and affordability. A Form3 SLA Printer (the successor of the Form2 used in this study) can be acquired for 3500 euros, tremendously cheaper than the infrastructure cost of a cleanroom. Furthermore, entry-level mSLA 3D printers are available for around 300 euros; while their quality might not be able keep up with the more expensive machines, they might still be useful for applications with larger dimensions. The vacuum former itself can be acquired for about 100 euros. The complete process can be performed without a cleanroom and with minimal training, as an entry-level vacuum former works with only a lever that moves the heated thermoplastic over the substrate and two buttons to turn the heating and vacuum on and off; the vacuum automatically carries out all the alignment of the thermoplastic. The use of an low-cost vacuum former, of course, has downsides due to its fixed heating and vacuum settings and fewer control points compared to more complex machines, like the ones made for microthermoforming [32]; however, the device makes up for it with its lower complexity, ease of use, availability and affordability, which might be especially beneficial for applications in low-income countries, where missing infrastructure can be a large bottleneck to research and the deployment of microfluidic devices, e.g., in point-of-care diagnostics [33]. To add to this, highly expensive devices might simply not be necessary in the first place, given that the aim of the method is to seal the microchannels with a relatively thick layer of thermoplastic. This is less prone to errors compared to creating said microchannels with a much thinner sheet of thermoplastic and higher pressure, such as for use in microthermoforming, which demands more stringent parameter control. Thus, easy to use and cheap devices might have the advantage for this application. Of course, further studies should take a closer look at all given parameters, like the thermoplastic used, the heating and the pressure difference applied (both as negative and positive pressure). However, for this, a new vacuum former needs to be constructed, which was beyond the scope of this study. Here, we have presented a proof-of-concept of the method and showed that the new technology provides an improvement over old monoplanar microfluidics, in terms of price, ease of use and achievable form-factors. Using 3D-printed substrates still results in relatively large channels, due to the limitation of current 3D-printing technology; however, topographic vacuum forming can also be applied to substrates manufactured by other means such as micro-milling, injection molding or hot embossing. This flexibility also enables this new technique to be used with a multitude of different materials, besides the resin-printed structures that are used. For example, thermoplastic substrates may enable better bonding and can create structures that are completely made from only one material. Metals, on the other hand, may be micro-milled and used as efficient heat exchangers or electrodes. Another remaining downside is the adhesion layer that still has to be applied to ensure leakage-free operation. While the use of spray-glue might not be a problem for many experiments, it can present a point of error in others, for example, in cell culture applications. While in this proof-of-concept study, a commercial all-round adhesive was used, it may also be possible to use biocompatible adhesives with known properties, applied via airbrush systems. Airbrush application of uncured polyurethane, for example, was carried out by our group for other research and could be used as a bonding layer. Another way in which this might be improved is the use of different thermoplastic sheets, as the thermoplastic we used was designed for molds and was intended to be non-stick. Finetuning of the factors discussed above—heating temperature, vacuums strength and the thermoplastic and substrate material used—might tremendously improve the results and make the use of an adhesive layer redundant.

While there is still room for optimization, this proof-of-concept for topographical vacuum forming already shows the great benefits it can bring to microfluidic manufacturing. In particular, its ease of use, affordability, cleanroom-free fabrication, fast turnaround time and the possibilities for highly advanced form factors make this technology highly attractive to microfluidic researchers, especially given the rising interest in 3D-printed microfluidics. Further developments in 3D printing will go hand in hand with Tovas and increase its area of application since Tovas is compatible with common 3D-printing techniques [34,35]. This is especially interesting since 3D printing is expanding increasingly into areas other than simple structure fabrication. The wide range of materials available for 3D printing can be used in colorimetric biosensor applications, as demonstrated in this study, but also in other potential biosensor applications, for example, in flow cells for fluorescent or electrochemical detection [36,37,38]. In addition to biosensing, these new 3D-printing materials can also form the basis of smart surfaces that serve additional functions [39,40]. Tovas can be a critical component to support this development and make 3D-printed microfluidics easier, cheaper and more available. The low barrier of entry will enable laboratories that are new to microfluidic-manufacturing to construct highly advanced microfluidic devices. Therefore, the presented method is attractive for both old and new microfluidic researchers.

## Supplementary Materials

The following are available online at https://www.mdpi.com/article/10.3390/bios11100395/s1, Figure S1: Detailed graph of the macro-structure alignment shown in Figure S5; Figure S2: Microscope images of the PDMS negatives to measure channel intrusion; Figure S3: Image and description of the vacuum former used; Figure S4: Microscopic image of the adhesive layer; Figure S5: Profile of the adhesive layer; Figure S6: Complex modulus and tan δ of the thermoplastic as a function of temperature; Figure S7: Storage Modulus E’ and Loss Modulus E’’ as a function of temperature; Figure S8: Microscopic image of a 3D-printed angle; Figure S9: Absorbance spectrum of the Polyethylene Terephthalate Glycol sheet.
