# Supplementary material for: Topographical Vacuum Sealing of 3D-Printed Multiplanar Microfluidic Structures
Source: Biosensors (Basel). 2021 Oct 15;11(10):395. doi: 10.3390/bios11100395 (PMC8534087; doi:10.3390/bios11100395)
Supplement: Supplementary file 1 [file biosensors-11-00395-s001.zip › biosensors-1400971-supplementary.pdf]

biosensors

Supporting Information

Topographical Vacuum Sealing of 3D-Printed Multiplanar Microfluidic Structures

Benjamin Heidt [1,*], Renato Rogosic [1], Nils Leoné [2], Eduardo J.S. Brás [3], Thomas J. Cleij [1], Jules A.W. Harings [2], Hanne Diliën [1], Kasper Eersels [1] and Bart van Grinsven [1]

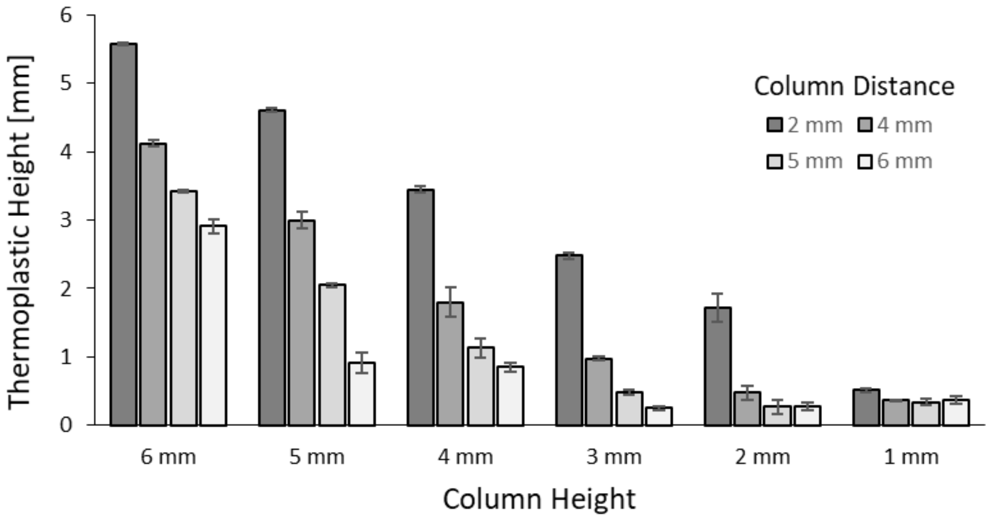

Figure S1. Detailed graph of the macro-structure alignment shown in Figure 5.

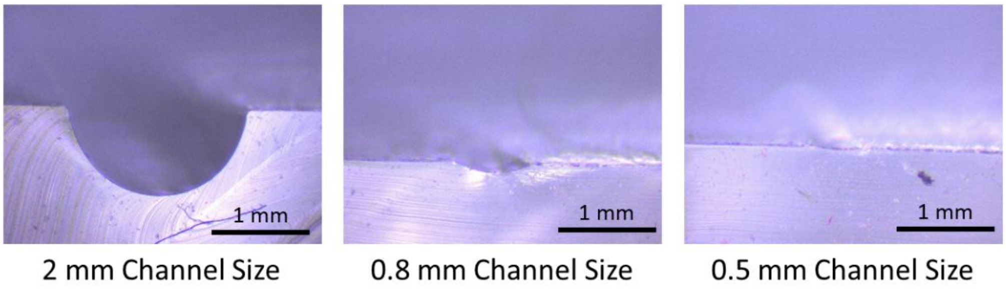

Figure S2. Example images of the PDMS negatives to measure channel intrusion, pictures display the intrusion into channels with sizes 2, 0.8 and 0.5 mm (left to right).

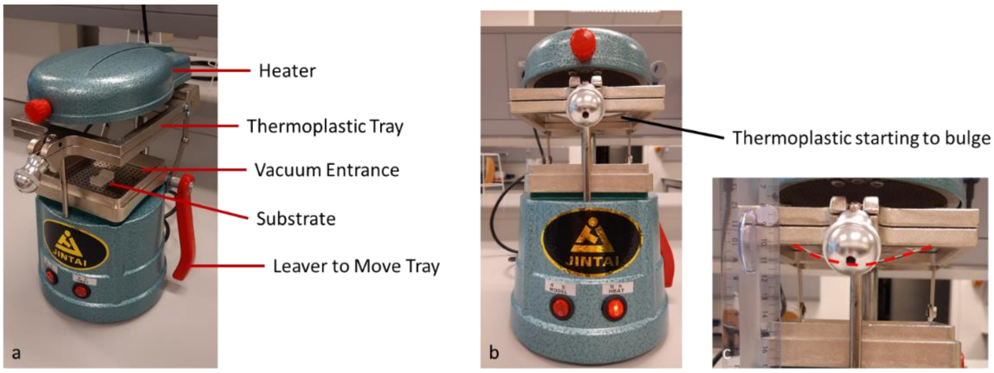

Figure S3. Low-cost vacuum former (a). The thermoplastic tray is moved under the heater and heated until the thermoplastic deforms and its bulge reaches 1 cm under the tray (b,c). The tray is then lowered over the substrate using the lever and the vacuum pump activated.

Adhesive Layer

An adhesive layer was applied to a glass slide to observe and measure its distribution and profile. Figure S4 shows a microscopic image of the adhesive layer (without added thermoplastic) and Figure S5 shows its profile. The surface was measured with a DektakXT (Bruker) equipped with a stylus of 2 µm radius and a force of 3 mg. The layer showed an average height of 1.75 µm with a maximum of 7 µm. Measurement directly on the 3D printed surface was difficult, however, as the print showed a similar roughness (2.22 µm) to the adhesive layer and no clear step was visible.

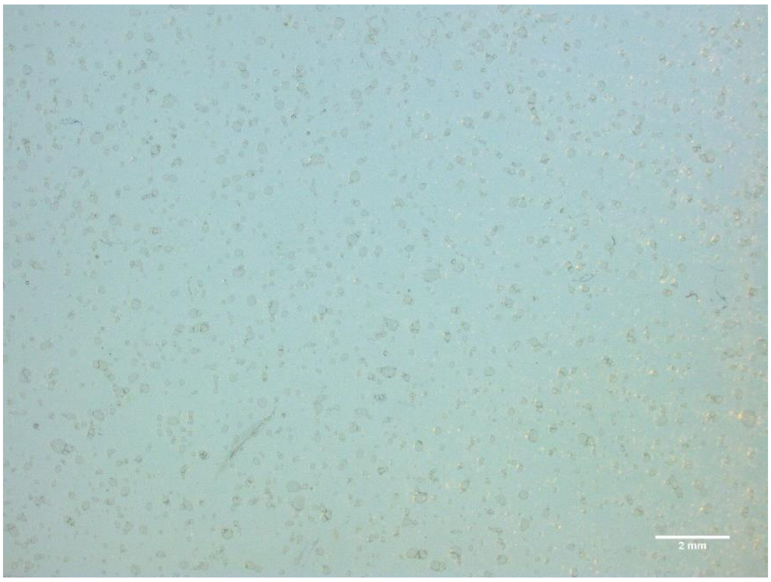

Figure S4. Distribution of spray adhesive after application on a glass slide and without the application of the thermoplastic.

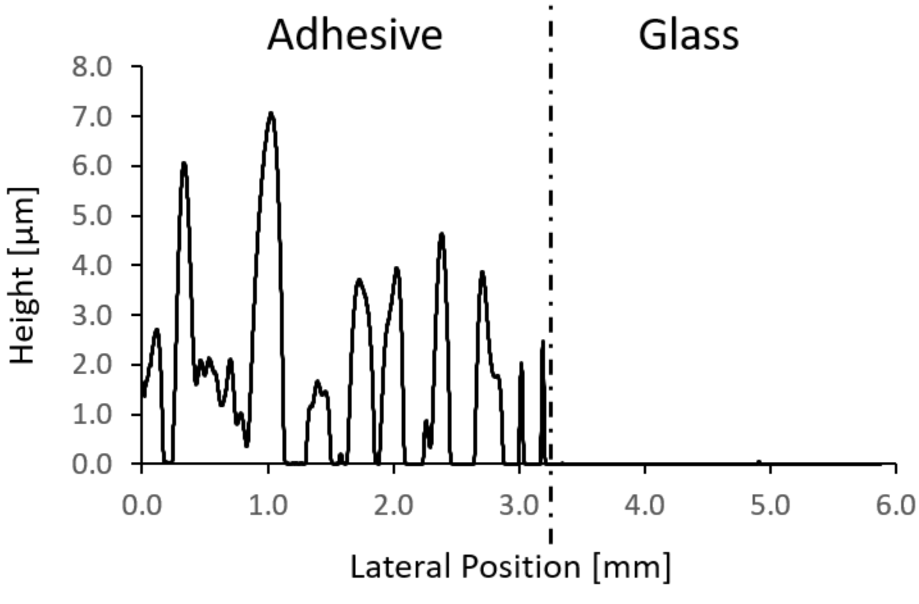

Figure S5. Profile of the adhesive layer on a glass slide.

Thermoplastic Characteristics

Dynamic mechanical analysis was performed with a DMA1 (Mettler Toledo) with a heating rate of 3 °C per minute, displacement of 10 μm/s and frequency of 1 Hz. Figure S6 shows the complex modulus and tan δ. Figure S7 displays the storage and loss modulus.

Tan δ displays two maxima, the first one being the glass transition temperature at 75 °C and the second one the sagging temperature at 140 °C.

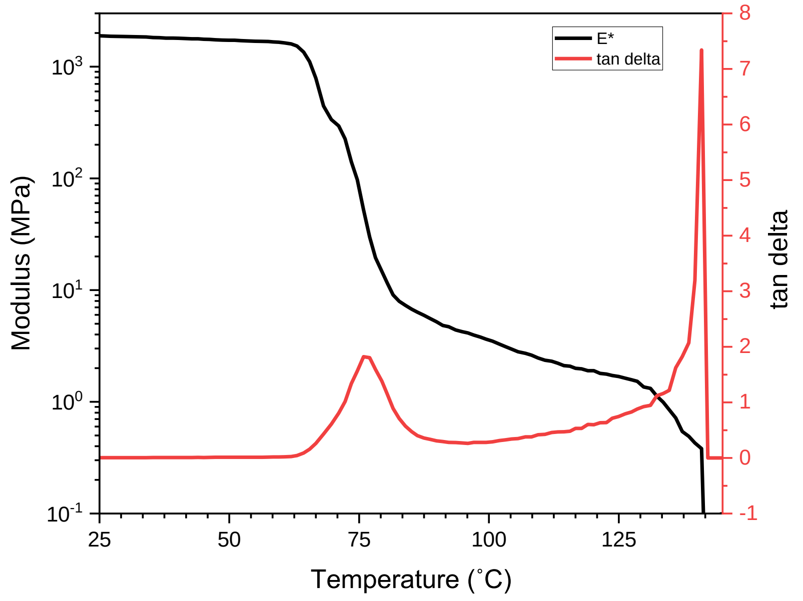

Figure S6. Complex modulus and tan δ as a function of temperature.

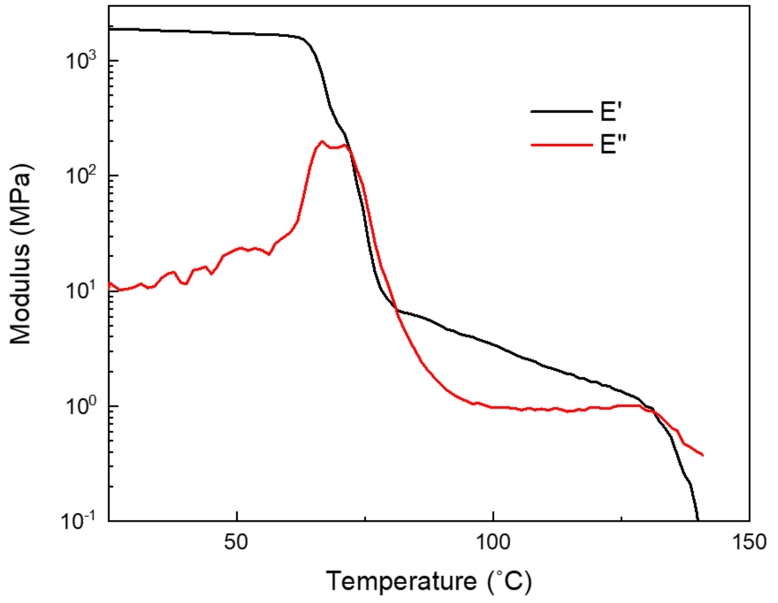

Figure S7. Storage Modulus E′ and Loss Modulus E′′ as a function of temperature.

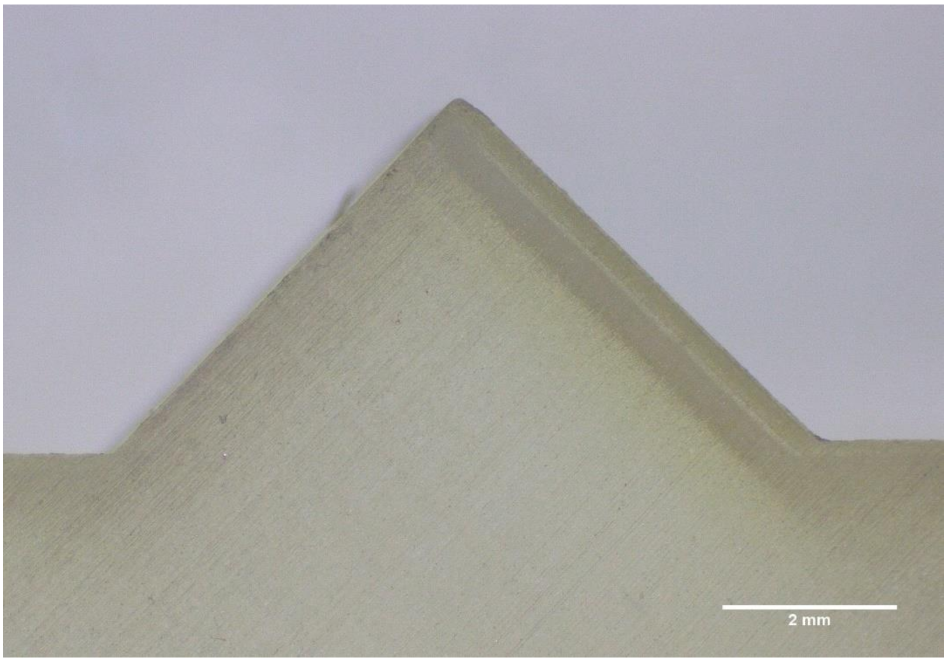

Figure S8. Image depicting the angle sharpness (angles: 135° and 270°) of a 3D print before sealing.

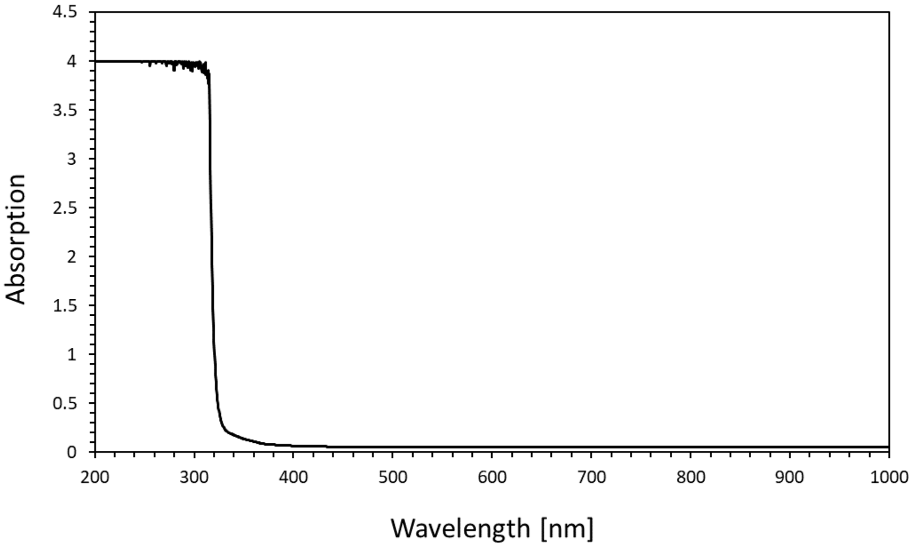

Figure S9. Absorbance spectrum of the Polyethylene Terephthalate Glycol sheet, acquired with a Shimadzu 1900i and a resolution of 0.5 nm.
